# Diaphragmatic Pleurisy

**Published:** 1893-11

**Authors:** 


DIAPHRAGMATIC PLEURISY.—W. S. Fenwick (*The Lancet*, July 3, 1803), reports five cases of pleurisy of the under surface of the lung, which closely simulated acute abdominal disease, and in which the diagnosis was made with difficulty:

Case I.—A man, aged 34, was suddenly seized with severe pain in the right hypochondrium. He was found leaning over a chair and suffering intensely. The superficial abdominal muscles were firmly contracted, and the least pressure over the hypochondrium or epigastrium was attended with the greatest suffering. The respiration was almost entirely costal and shallow. Expiration was accompanied by a grunt. The liver appeared to extend below the margin of the ribs. The temperature was about 103. No alteration occured until the third day, when dullness at the right base of the lungs was discovered. This sign rapidly developed until a large effusion could be demonstrated in the right base. With the physical signs of pleurisy, the case began to improve. The case was first diagnosed as inflamation of the gall-bladder.

Case II was addmitted with what was supposed to be perityphlitis. After bathing in the river he was suddenly seized with a chill and severe pain in the right iliac fossa. Vomiting was constant, with high fever. The bowels were confined. The ileo-cecal region was exceedingly tender. No tumor could be demonstrated. There were no chest signs. On the fourth morning a soft friction sound was heard over the right base. The case rapidly developed into one of pleurisy over the right lower lobe.

Case III.—An old phthisical case was suddenly seized with excruciating abdominal pain. The patient lay with knees drawn up, and was the picture of one with peretonitis. Death occurred from supposed peritonitis, but at the autopsy recent diaphragmatlc pleurisy was discovered, and the peritoneum was normal.

Case IV.—A boy was suddenly seized with pain in the abdomen and vomiting. On admission the temperature was 103, the pulse was full, the abdomen exquisitely tender to pressure. The case looked like one of peritonitis. On the fourth day chest signs appeared. A pleurisy of the left base developed, and the case improved with undoubted signs of effusion.

Case V closely resembled Case II, and recovered.

These abnormal signs, the author explains, upon the hypothesis of reflection along the line of the intercostal nerves, and manifestation of pain at extremities of the nerves. The pain is usually more superficial than that observed in diseases of the abdominal viscera, and is markedly increased by the respiratory movements. No tumor can be found, and rectal examination is negative. Many of the diagnoses can be determined only on the appearance of serious effusion at the base of the lung.—*University Medical Magazine.*

